# Light-Induced Neuron-Like Bursts

**DOI:** 10.1021/acs.jpclett.6c01968

**Published:** 2026-08-13

**Authors:** Jitendra Kumar, Roberto Fenollosa, Gonzalo Rivera-Sierra, So-Yeon Kim, Juan Bisquert

**Affiliations:** Instituto de Tecnología Química (ITQ), Consejo Superior de Investigaciones Científicas−Universitat Politècnica de València, 46022 Valencia, Spain

## Abstract

Aside from recent advances in artificial intelligence (AI) models, specialized AI hardware has become increasingly important for processing large volumes of unstructured and dynamically evolving data at the edge. The growing demand for on-device learning calls for dynamically reconfigurable systems that are capable of responding to continuously changing environments. Here, we demonstrate a dynamic approach to information processing using devices exhibiting negative differential resistance operated near their folding point, where oscillatory input signals induce controllable bursting dynamics. In this regime, time-varying input signals generate tunable spike responses governed by the waveform characteristics. We systematically investigate the influence of input amplitude, frequency, and DC offset on the resulting device dynamics. The observed sensitivity enables compact encoding and discrimination of temporal signal features without external clocking. These results establish negative differential resistance devices as promising platforms for asynchronous signal classification based on intrinsic nonlinear dynamics.

## Graphical abstract

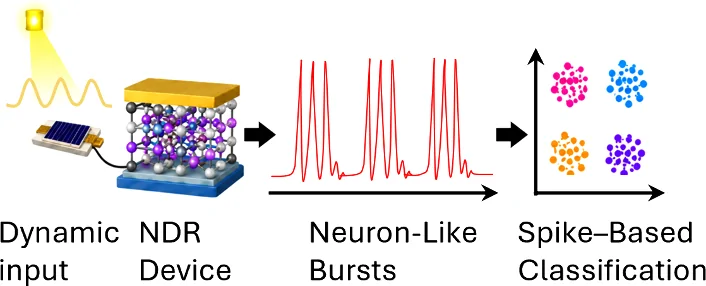

The rapid growth of artificial intelligence (AI) applications outside centralized computing environments is increasing the need for compact and energy-efficient hardware capable of processing complex temporal information directly at the edge.^1–7^ Conventional digital systems, typically based on synchronous clocked architectures, are not well-suited for sparse or irregular data streams, since computation proceeds independently of actual signal activity. By contrast, asynchronous approaches operate in an event-driven manner,^8–10^ enabling low-latency and power-efficient processing while simplifying the integration of heterogeneous components without clock synchronization constraints.^11–15^ Such adaptive architectures are particularly attractive for real-time edge intelligence operating in continuously changing environments.^1,3,16^

Among the emerging candidates for physics-based information processing, nonlinear electronic devices exhibiting negative differential resistance (NDR) provide rich intrinsic dynamics that can be directly exploited for computation.^17–19^ Because natural signals are inherently time-dependent, relevant information is often encoded in temporal features, such as frequency, amplitude, and waveform structure, rather than in static values alone. Consequently, understanding the response of spike-generating systems to oscillatory inputs is essential for temporal signal analysis. Here, we investigate a NDR device operated near the folding point of its current–voltage (*I*–*V*) characteristics, where time-dependent excitation induces controllable transitions between quiescent states and bursting spike activity.^17^ A state was classified as bursting when at least three consecutive spikes occurred with interspike intervals below 5 ms, separated from the next burst by a silent period of the same duration. Otherwise, the activity was classified as quiescent. The resulting dynamics exhibit strong sensitivity to the input amplitude, frequency, and DC offset, producing tunable spike patterns that enable compact encoding and discrimination of temporal signal features. These results demonstrate the potential of NDR-based dynamical systems for asynchronous neuromorphic signal classification without external clocking or complex control circuitry. In a practical signal classification framework, this system can operate as a front-end encoder, converting analog input signals into spike trains whose spike rate can be processed by downstream event-driven classifiers, such as spiking neural networks. Such an event-driven architecture eliminates the need for continuous sampling, thereby reducing latency and improving energy efficiency. Figure 1b–f shows the experimental bursting outputs generated by a thyristor-based NDR device; it is shown that the spike count per burst and the intraburst spiking frequency can be precisely controlled by modulating the input signal.

**Figure 1:**
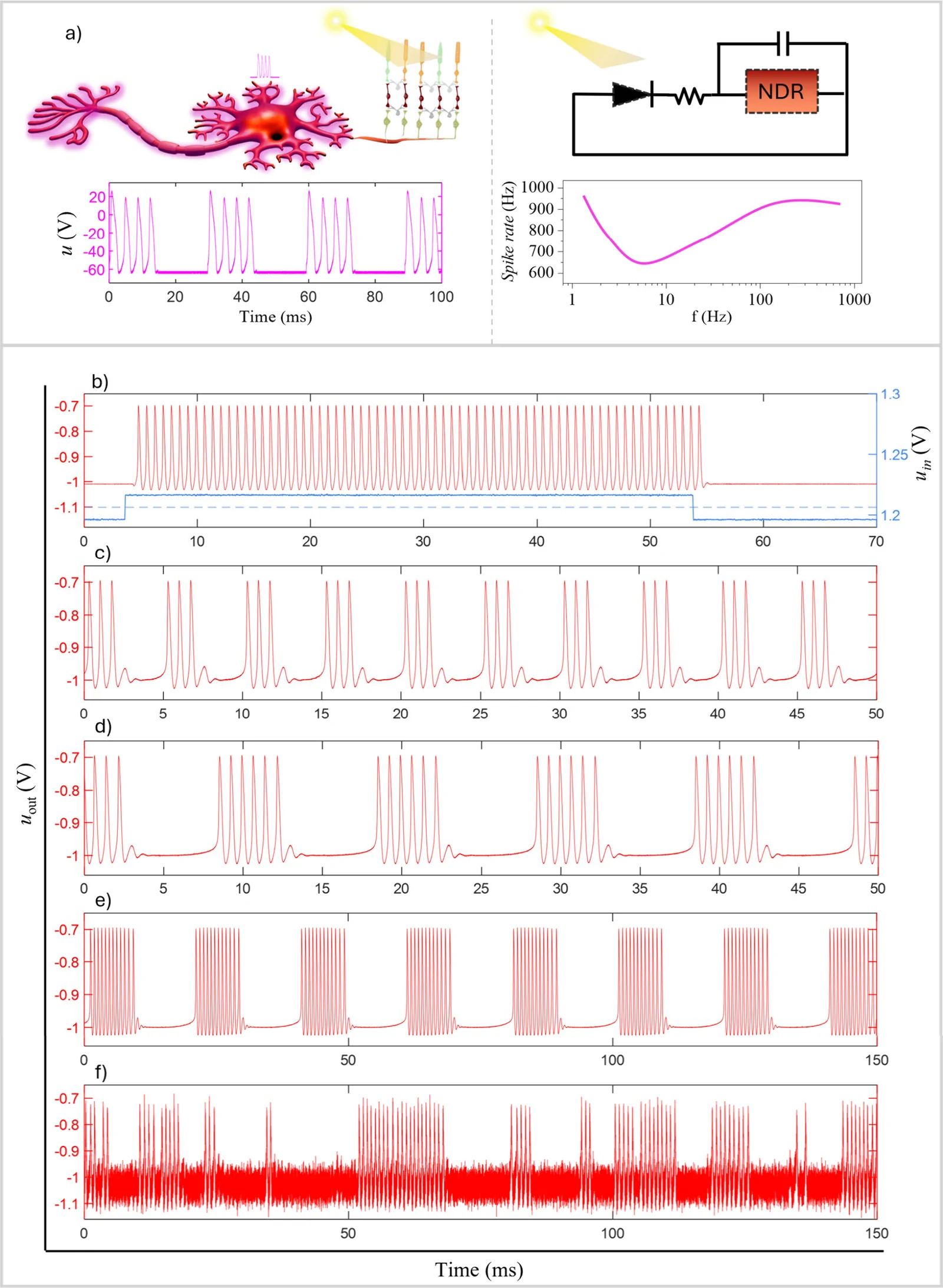
(a) Schematic illustration comparing a biological neuron and a NDR-device-based artificial neuron, (b−f) different oscillatory responses of the NDR device to different input signals (b) response to piecewise constant pulse waveform as input, (c−e) response to a sine-wave input of the form: *v*_in_(*t*) = 1.198 + 0.010 sin­(2π·*f*·*t*) V, (c) *f* = 200 Hz, (d) *f* = 100 Hz, (e) *f* = 50 Hz, (f) response to a noisy input with a constant DC voltage of amplitude 2.05 V modulated with white noise of amplitude 5V. [The sign of the output voltage (*u*_out_) is inverted in the plot for better visualization].

In our experiments, the externally injected current serves as the bifurcation parameter, dynamically driving the system across a Hopf bifurcation and inducing repeated transitions between equilibrium attraction and oscillatory limit cycle dynamics. The quiescent phase is governed by a stable fixed-point attractor (Figure 2b), whereas the active phase (bursting period) is characterized by a stable limit cycle (Figure 2a).

**Figure 2:**
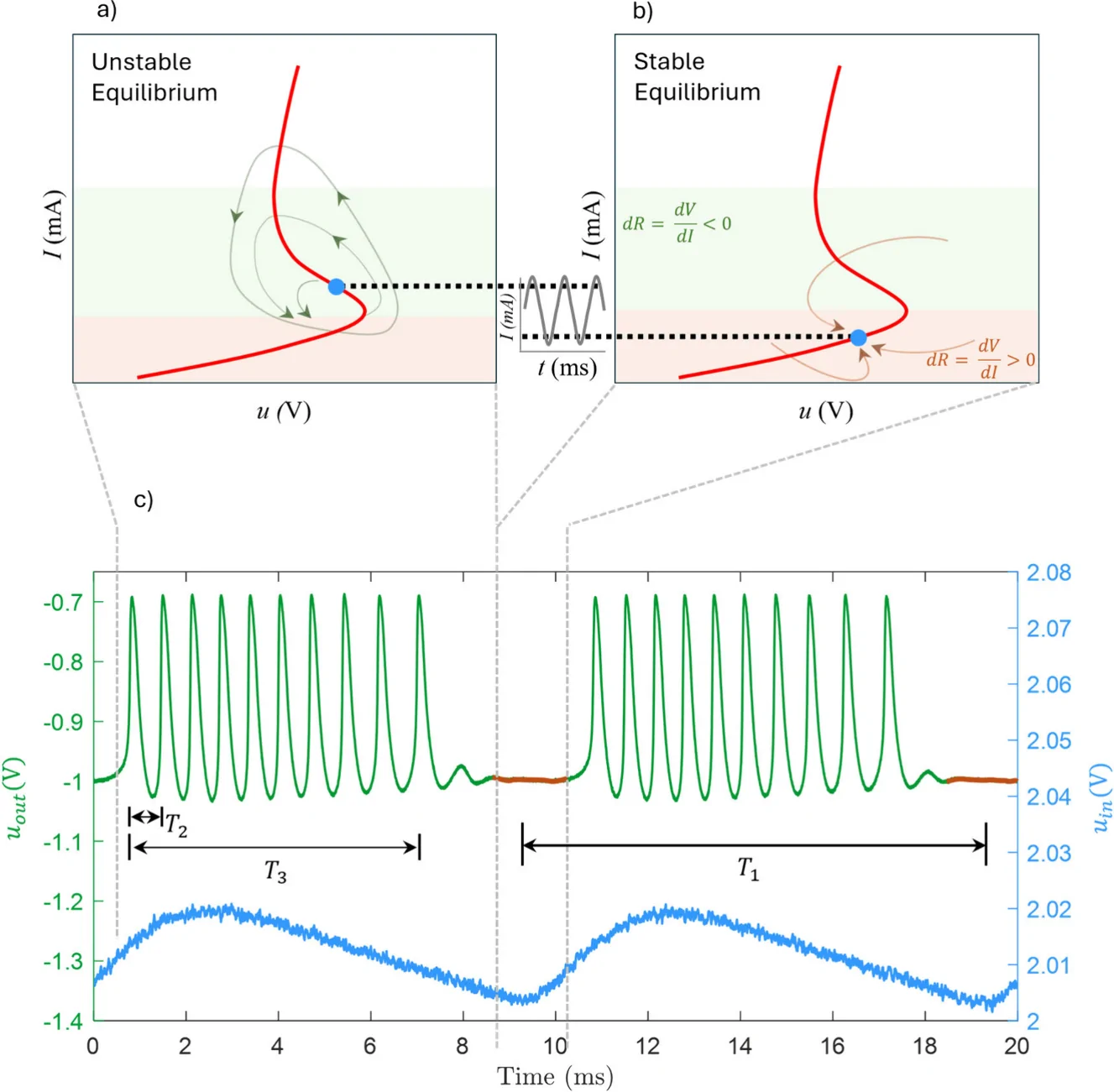
Schematic diagram of a phase portrait of a coupled thyristor–solar cell dynamical system. (a) Bifurcation parameter (input current) leads the system to the emergence of an unstable equilibrium point and a limit cycle. (b) Bifurcation parameter leads the system to the stable equilibrium state. (c) Experimental demonstration of the system transition between stable equilibrium and unstable equilibrium resulting in bifurcation and emergence of a limit cycle leading to neuron-like bursts.

This transition is mediated by a slowly time-varying current whose frequency, amplitude, and average DC level act as dynamic control parameters for sweeping the system across a critical bifurcation manifold. As the injected current increases and crosses the bifurcation threshold, the previously stable fixed point loses stability, forcing the trajectory to depart from equilibrium and relax to an emerging periodic limit cycle. When the current decreases below the bifurcation point, the periodic limit cycle vanishes and a stable attractor is restored, resulting in the quiescent phase of the oscillator. This rhythmic alternation between stationary and oscillatory states organizes the phase space into a bursting pattern (Figure 2c), in which the temporal evolution is determined by the repeated emergence and collapse of the limit cycle in response to the continuously shifting geometry of the underlying vector field.

The impact of input signal frequency (*f*_in_), amplitude (*A*_in_), and average DC level (*u*_in,avg_) was further investigated to assess the possibility of encoding information in both inter- and intraburst frequency by monitoring the number of spikes per unit time. The spike rate *r*, defined as the number of spikes per second, can be measured asynchronously to encode or extract information from the burst patterns. We found that the spike rate depends not only on the input signal frequency (Figure 3a) but also on the signal amplitude (Figure 3b) and average DC level (Figure 3c and d). This demonstrates that the spike rate is sensitive to multiple characteristics of the input signal, making it a useful parameter for encoding time-dependent signals in an asynchronous manner.

**Figure 3:**
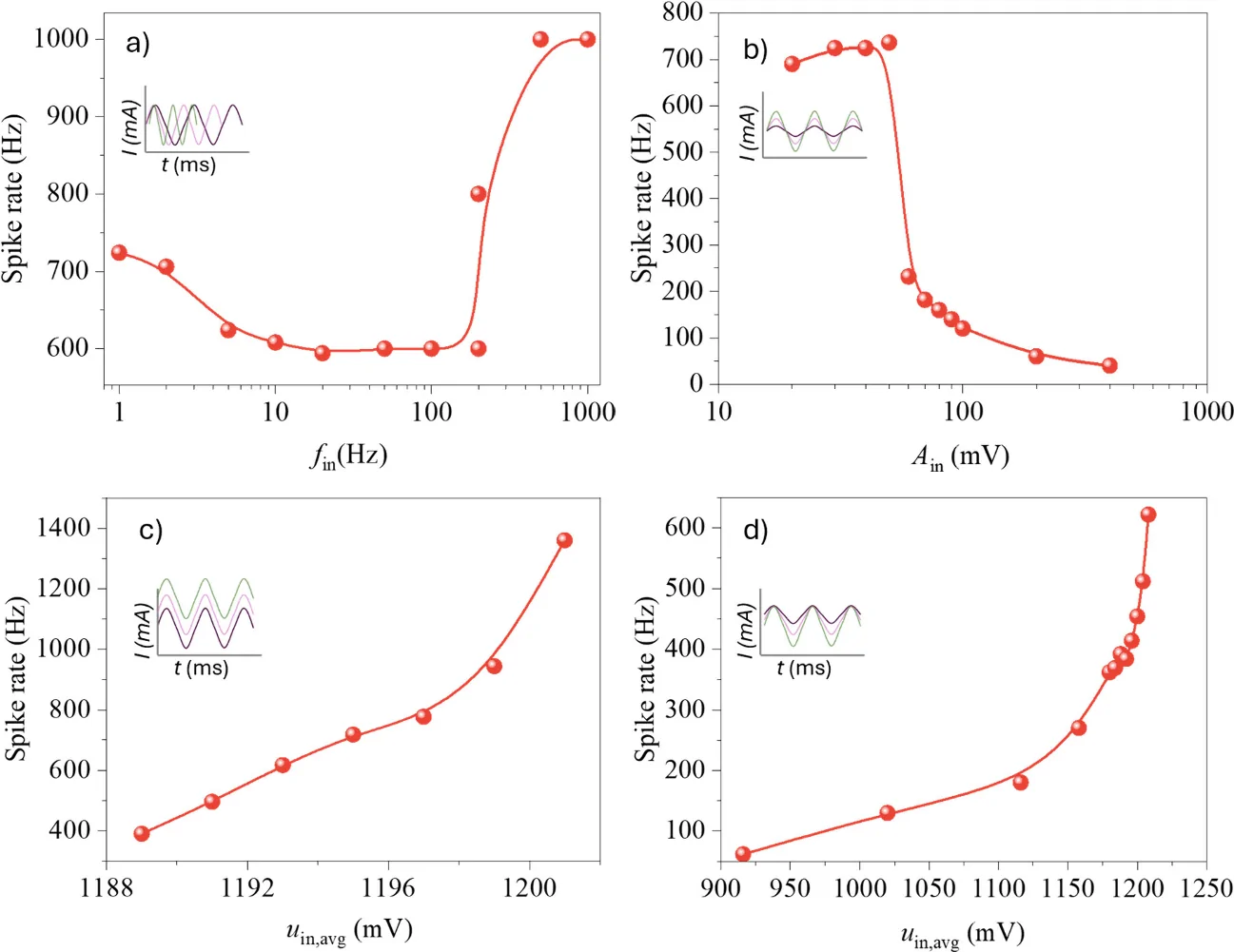
Demonstration of precise controllability of the spike rate under different input conditions. (a) Input frequency vs spike rate. (b) Input voltage amplitude vs spike rate. (c) DC offset of the input voltage is varied while keeping the amplitude of the sinusoidal voltage waveform constant. (d) DC offset of the input voltage is varied while keeping the peak voltage constant.

With the increase of the input signal frequency from very low to high frequency, the spike rate tends to decrease in the beginning; thereafter, it rapidly increases and aligns with the frequency of the input, as the input frequency approaches the intraburst frequency (Figure 3a). The dependence of the spike rate vs input signal frequency is governed by the interplay of the relaxation time constant for the current and parallel capacitance of the thyristor. As the frequency of the input signal increases, the system does not have enough time to move itself from the basin of attraction around a stable point to the basin of attraction around a limit cycle. This is reflected as the decreased spike rate, whereas when the frequency of the input start to approach that of the intraburst frequency of the oscillations, the spike rate again increases. The matched input and intraburst frequency create a special synchronization condition. The peaks of the input align with the oscillator phase that lies in the basin of attraction of the limit cycle. The valleys of the input align with the oscillator phase that lies in the basin of attraction of the stable point. Thus, it becomes easy for the oscillator to follow the input, and therefore, increasing the input frequency leads to an increased spike rate.

The amplitude of the input signal also affects the spike rate response of the system, and this dependence is easier to understand. As the amplitude increases, the system has more time to spend in the unstable equilibrium, which leads to the increased spike rate for low to mid-input amplitudes (Figure 3b). With a continued increase in the amplitude of the input voltage, the systems not only switches from stable to unstable equilibrium and back, but now there exist two stable equilibrium states. First, when the input current sets the voltage nullcline below the folding point of the thyristor, another stable equilibrium exists where the injected current sets the voltage nullcline above the negative differential resistance regime of the thyristor. The existence of two stable equilibrium states is clearly evident in the voltage–time traces measured as a function of the input voltage amplitude (shown in Figure S4). Thus, spikes are generated only for a part of the rising and falling edges of the oscillatory input current. Once the input current starts to drive the system from one stable equilibrium to another stable equilibrium, a further increase in the injected current is only going to increase the fraction of time when the oscillator is in stable equilibrium. This is reflected as the monotonic decrease of the spike rate after 50 mV of input voltage.

Increasing the average input voltage also leads to a monotonic increase in the spike rate because it increases the fraction of time that the system spends in the oscillatory regime relative to the time spent in stable equilibrium. The average input can be increased in two different ways: first, by keeping the signal amplitude constant while increasing the DC component and, second, by keeping the peak signal fixed while increasing the DC component. Distinguishing between these two cases is useful for identifying the shape of the incoming signals. We observed that the spike rate responds substantially differently in these cases, exhibiting a significantly larger dynamic range in the latter case. This allows clear distinction between two cases.

Bursting responses are highly valuable in spiking-neural-network-based and neuromorphic computing. Bursts can convey richer temporal information than isolated spikes while improving the robustness and reliability of the neural communication. Since synaptic transmission is probabilistic, multiple spikes within a burst increase the likelihood of successful downstream activation. Variations in spike timing, burst duration, and intraburst firing rate also enable selective signaling and multiplexed information encoding. Beyond reliable transmission, bursts can support a wide range of computational functions in artificial neural systems. Bursts can enhance attention and saliency detection, synchronize distributed neural activity, and filter noise.

In conclusion, we demonstrated that operating the negative differential resistance devices near the folding point and applying a time-varying input signal enable controlled bursting responses. We also studied the device response with respect to the input signal amplitude, frequency, and average DC offsets. We found that the number of spikes per second depends on all of these input features, making this platform well-suited for asynchronous signal classification systems.

## Methods

### Characterization for a Coupled Thyristor–Solar Cell System

To feed the oscillatory current as input, a voltage waveform generator in series with an input resistor of 470 Ω or a solar cell module is used. A 50 nF capacitor was used in parallel with the thyristor to observe the oscillations/spikes. Voltage-versus-time traces are measured using an oscilloscope (Picoscope 4262) from Pico Technologies. The output voltage is measured across the capacitor connected between the anode and the cathode of the thyristor. The input voltage is measured across the nodes where the thyristor is intended to be connected, with both the thyristor and the capacitor removed from the circuit. Since the input and output voltages are measured independently as two different measurements, the phase difference between the input and output is only indicative and not exact.

Since our system produces spikes with an average intraspike frequency of approximately 1500 Hz and is capable of generating self-sustained oscillations for a given DC input, we varied the input frequency from 1 Hz up to values approaching the average intraspike frequency. As the input frequency approaches the intraspike frequency, the system produces fewer spikes per burst, while the overall spike rate increases. Beyond this point, the activity started to fail to satisfy the criterion of bursting.

The input amplitude is selected in such a way that the voltage range during the quiescent phase remains below the voltage range associated with the bursting phase.

The chosen range of the DC offset also depends on the chosen input amplitude. However, the range of DC offset is to be selected such that the peak input voltage crosses the bifurcation threshold during one part of the input cycle and remains below it during another part of the cycle, i.e., [*u*_in,peak_ > *u*_in,bifurcation_ and *u*_in,valley_ < *u*_in,bifurcation_], where *u*_in,bifurcation_ is obtained from the steady-state *I*–*V* characteristics and corresponds to the fold (turning) point voltage. Under these conditions, the system alternates between the quiescent and self-oscillatory regimes, thereby producing bursting behavior.

## Supplementary Material



## Data Availability

The data presented here can be accessed at 10.5281/zenodo.20440340 (Zenodo) under the license CC BY 4.0 (Creative Commons Attribution 4.0 International).

## Notes

Current-voltage characteristics of the thyristor and solar cell module, variation of intraburst frequency, input frequency, and amplitude, and DC offset dependence of the spike rate (PDF)
