# Supplementary material for: Light-Induced Neuron-Like Bursts
Source: J Phys Chem Lett. 2026 Aug 13;17(34):9856–61. doi: 10.1021/acs.jpclett.6c01968 (PMC13528274; doi:10.1021/acs.jpclett.6c01968)
Supplement: Supplementary file 1 [file jz6c01968_si_001.pdf]

Supplementary Materials for

Light-Induced Neuron-Like Bursts

Jitendra Kumar[1,], Roberto Fenollosa[1], Gonzalo Rivera-Sierra[1], So-Yeon Kim[1], Juan Bisquert[1,*]*

[1]Instituto de Tecnología Química (ITQ), Consejo Superior de Investigaciones Científicas-Universitat Politècnica de València, 46022, Valencia, Spain.

*Corresponding authors E-mail: jkumar2@itq.upv.es, jbisquer@itq.upv.es

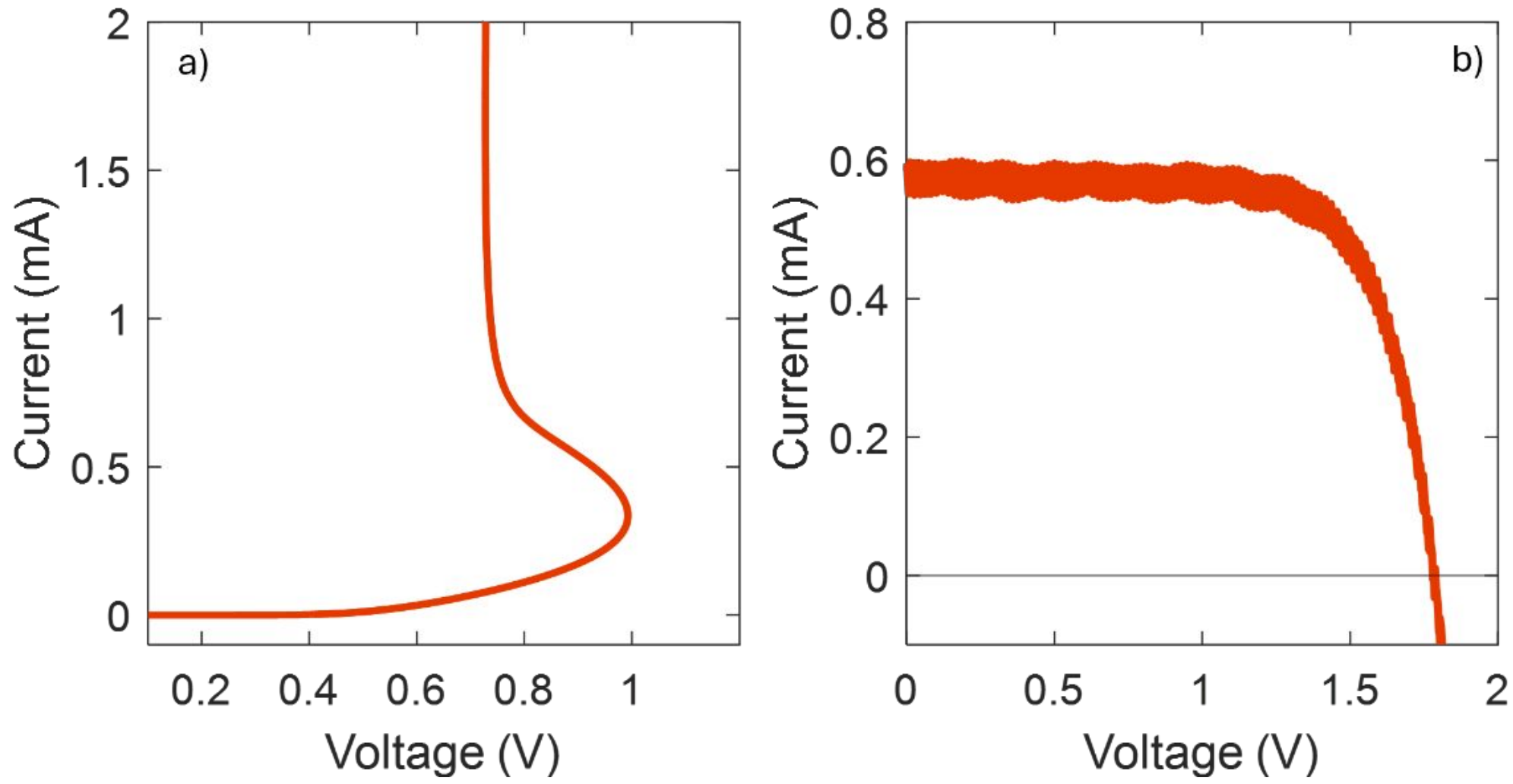

Figure S1. Current voltage characteristics of a) thyristor measured in galvanostatic mode, b) of a module of solar cells.

Figure S1a, and S1b shows the current voltage characteristics (IV) of thyristor and solar cell respectively. Thyristor IV shows S-type negative differential resistance with a the folding current and peak point voltage within the range of the current and voltage provided by the solar cell module. It is to be noted that the intensity of the required illumination depends on the size of the module and therefore it not critical parameter for our study.

Figure S2 shows that the intra-burst spike frequency (f_{out}), can be tuned by adjusting the input signal frequency f_{in}. This demonstrates that the system allows control over not only the spike rate and the inter-burst frequency, but also intra-burst spike frequency directly. It is important to note that controlling spike rate alone does not necessarily imply the tunability of f_{out} however, achieving control over f_{out} is sufficient to already claim that spike rate is also controlled.

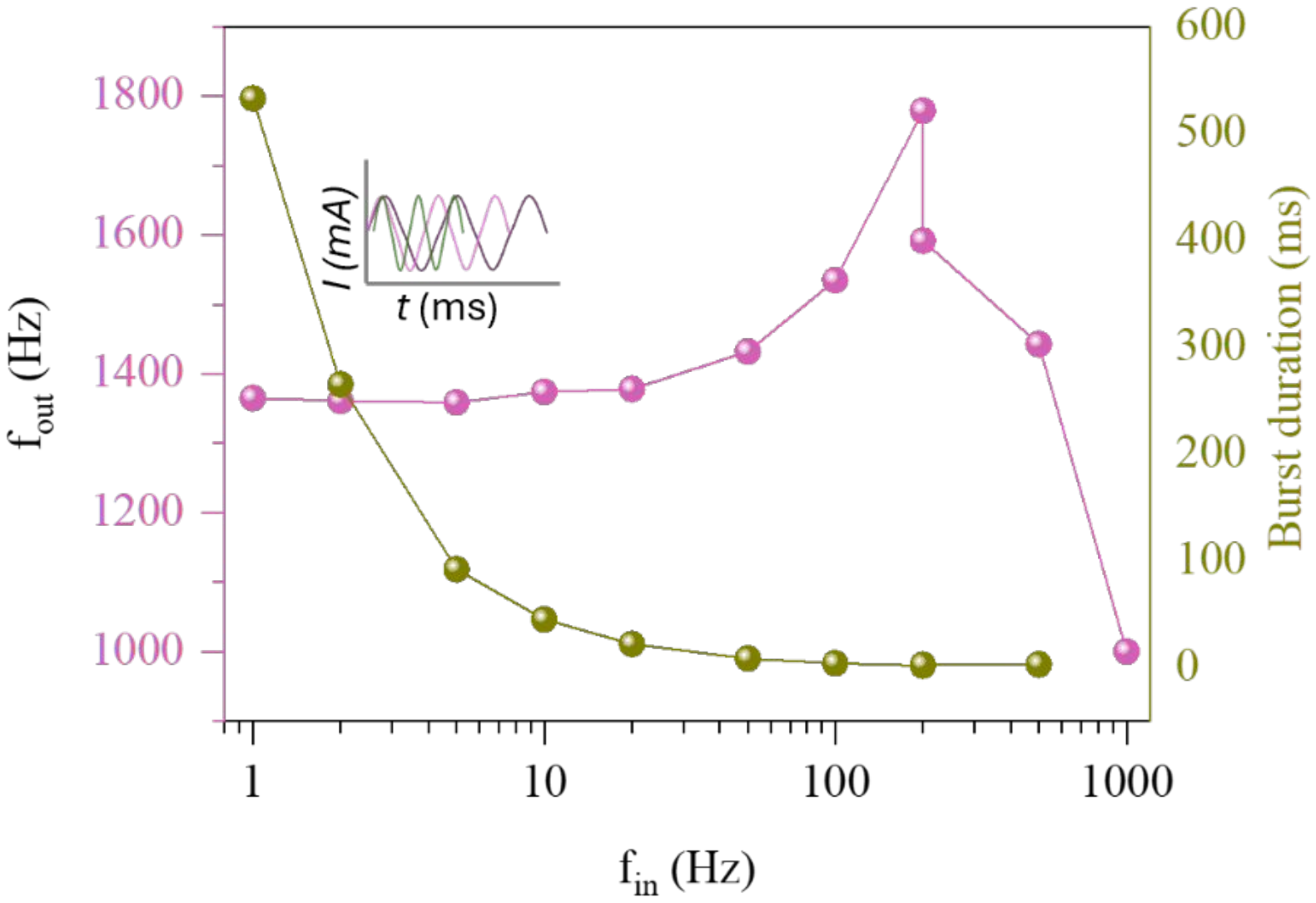

Figure S2. Demonstration of controllability of the intra-burst spike frequency.

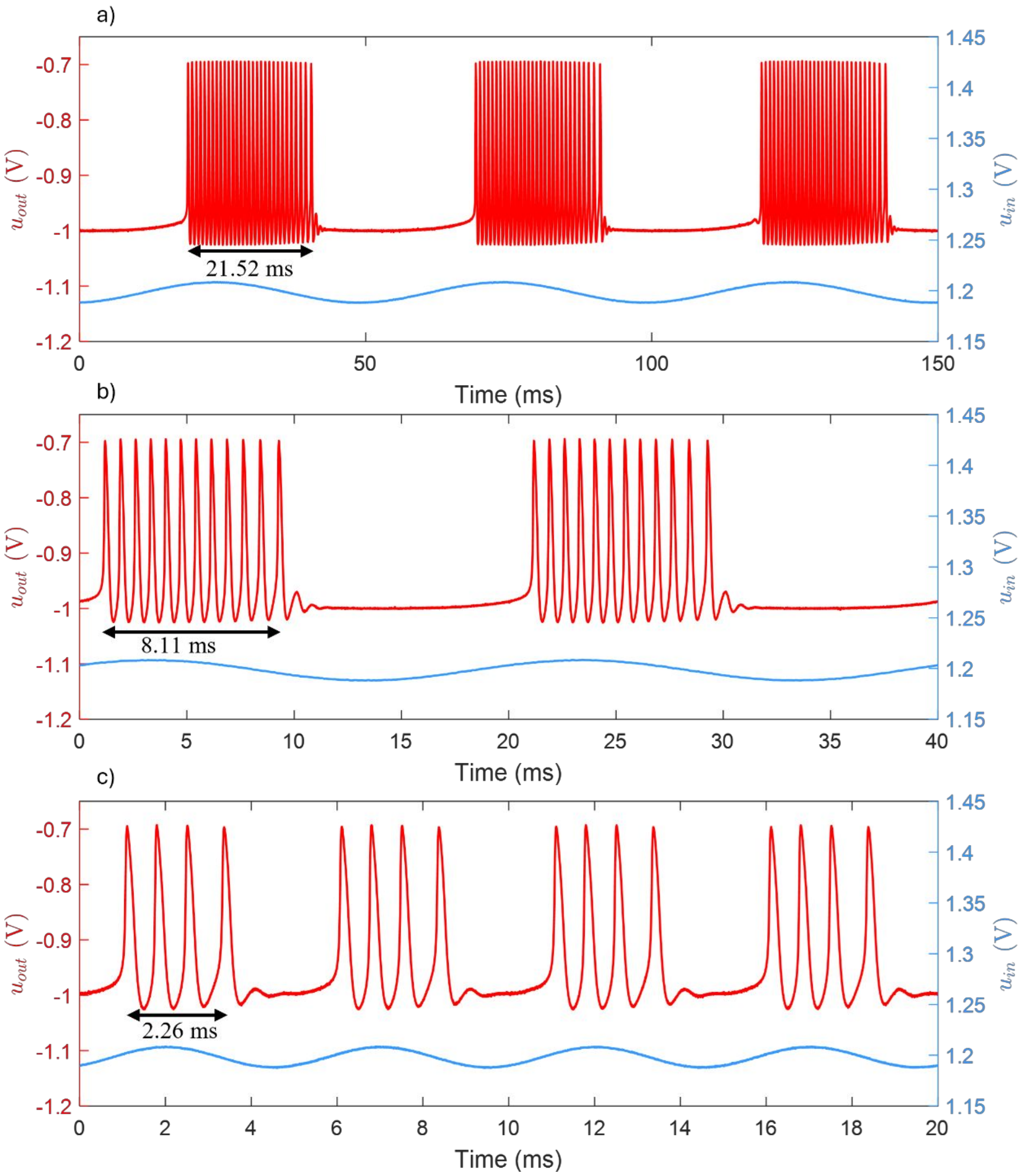

Figure S3. Modulation of spike rate, inter-burst and intra-burst frequency by varying the input signal frequency.

As observed from Figure S3, tuning the frequency of the input signal, both inter-burst, and intra-burst frequency can be tuned. As a result, the spike rate becomes input frequency dependent.

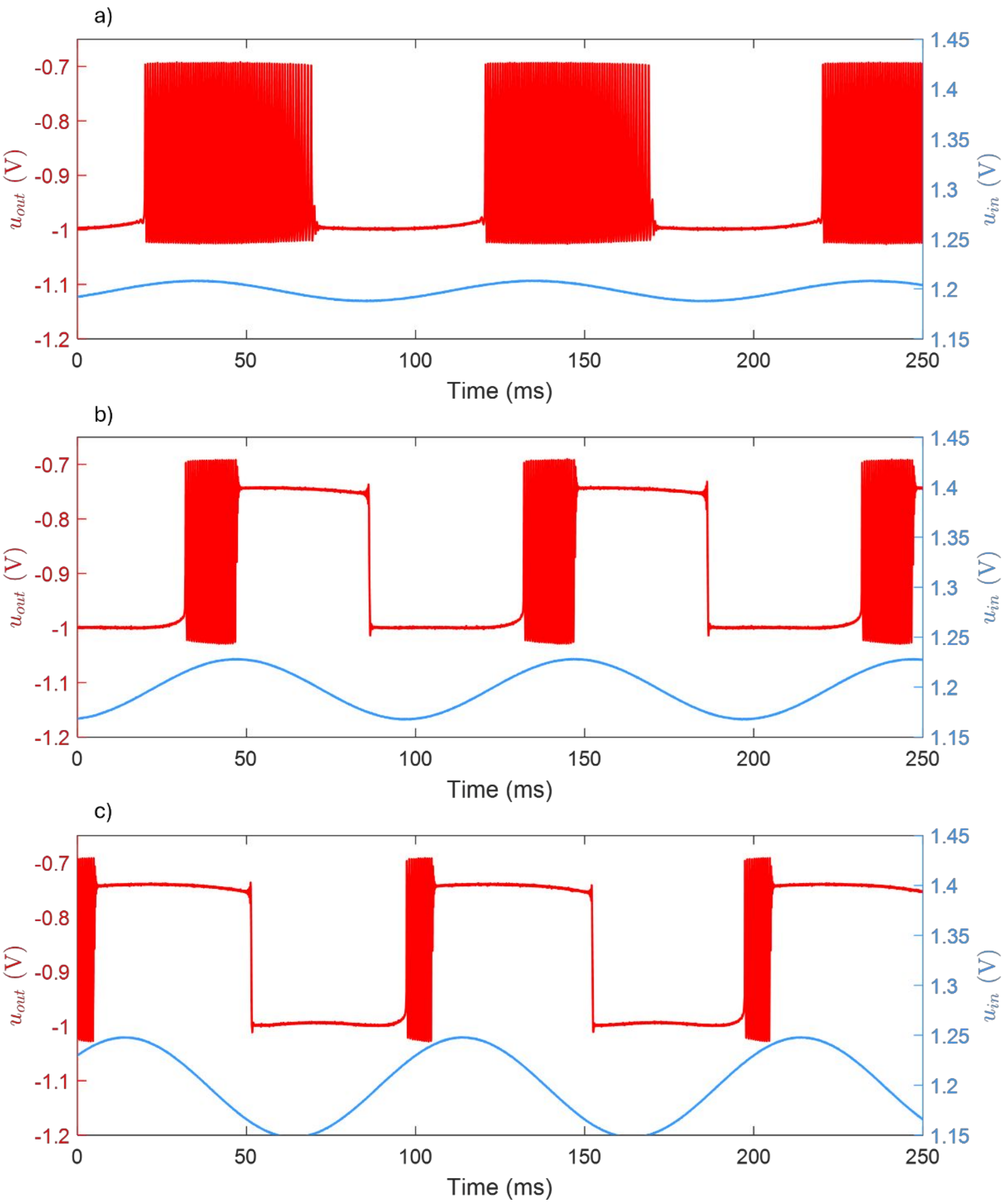

Figure S4. Modulation of spike rate by tuning the amplitude of the input voltage.

Input signal amplitude also affects the spike rate; by affecting the time the system spends in the basin of attraction of the stable equilibrium vs in the time it spends in the basin of attraction influence of the oscillatory limit cycle, (Figure S4). At higher amplitudes of the input voltage, the operating point moves between one stable equilibrium

to another stable equilibrium (Figure S4b,c).

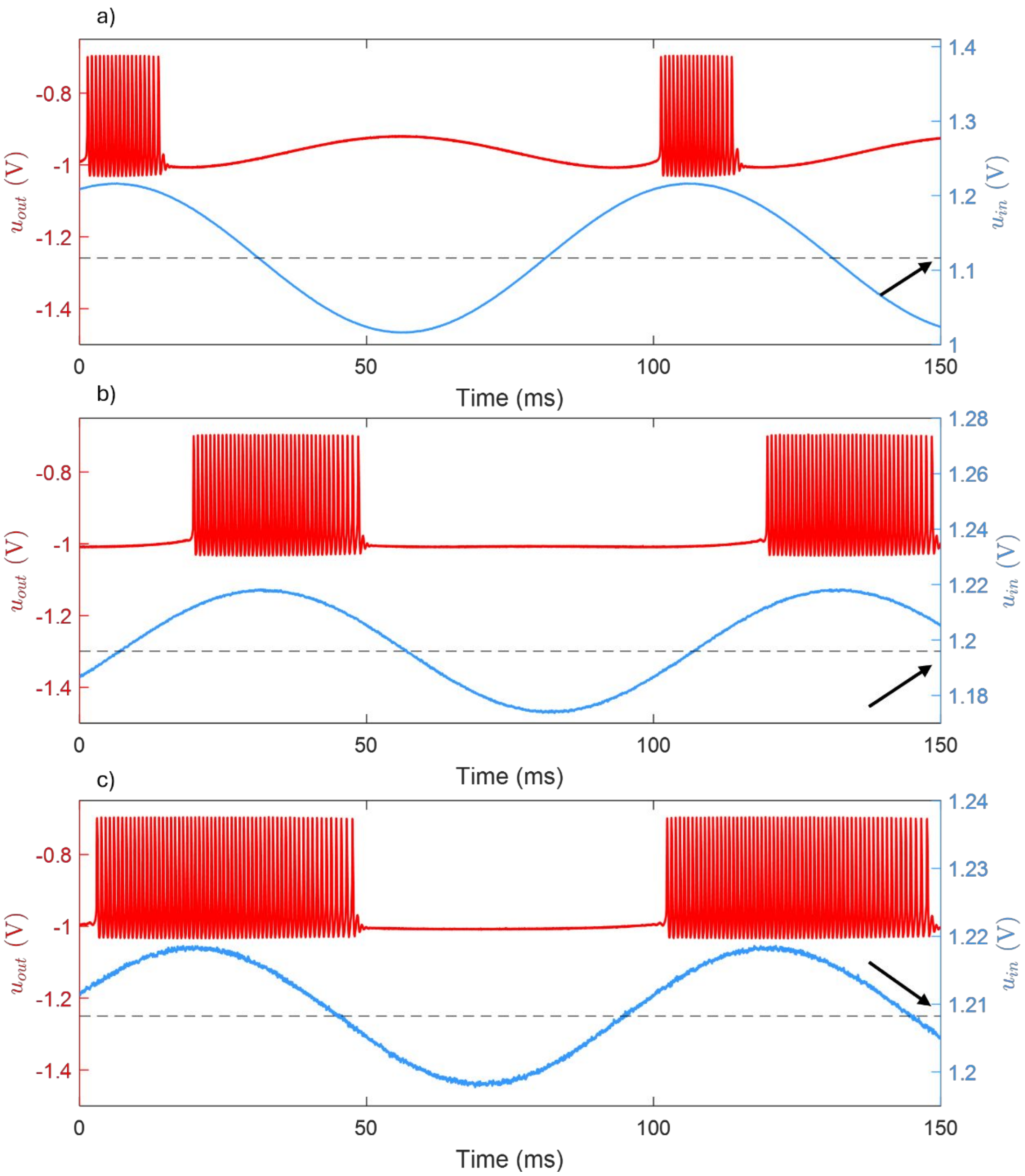

Figure S5. Spike rate tunability by tuning the average (offset) of the input voltage, keeping the peak input voltage constant.

Figure S5, shows that a change in DC offset of the input voltage, lets us tune the duty cycle of the bursts, which clearly shows that the spike rate is sensitive to the input signal amplitude. Thus, average DC offset behaves as a control parameter of the spike rate.

Mathematical model of light induced neuron like bursting

The dynamics of the photovoltaic thyristor oscillator are described by the following nonlinear system:

$$\frac{du}{dt} = \frac{1}{C_0}[I_{solar}(u,t) - g(x)u] \tag{1}$$

$$\frac{dx}{dt} = \frac{1}{\tau_k}[g(x)u - x] \tag{2}$$

The time-dependent photocurrent is given by

$$I_{ph}(t) = f(t) \tag{3}$$

The nonlinear conductance of the thyristor is defined as

$$g(x) = \frac{x}{U_{eq}(x)}$$

and the equilibrium current – voltage current–voltage characteristic is

$$U_{eq}(x) = R_v x + V_0 ln\left|\frac{d_0^2 a_0 + d_0 a_1 x + a_2 x^2}{d_0 b_0 + b_1 x + b_2 x^2}\right| + V_1 \tag{4}$$

The photovoltaic current follows the Shockley diode equation,

$$I_{solar}(u,t) = I_{ph}(t) - I_{0,solar}(e^{au} - 1) \tag{5}$$

Where,
C_0 is capacitance, τ_k is current relaxation time, R_v, V_0, V_1, d_0, a_1, a_2, b_0, b_1, b_2 are thyristor fitting parameters.
I_0 is maximum photocurrent, $I_{0,solar}$ is solar cell saturation current, $a = \frac{1}{\eta_f k_b T}$ is diode exponential coefficient , $f(t)$ is time dependent photocurrent.

The Jacobian of the dynamical system is written as,

$$J = \begin{bmatrix} (-a \cdot I_{0,solar}(e^{au} - 1) - g)/C_0 & -(u/C_0) \cdot g' \\ g/\tau_k & (u \cdot g' - 1)/ \end{bmatrix}$$

$$g' = (U_{eq} - x \cdot U'_{eq})/U_{eq}^2 \tag{6}$$

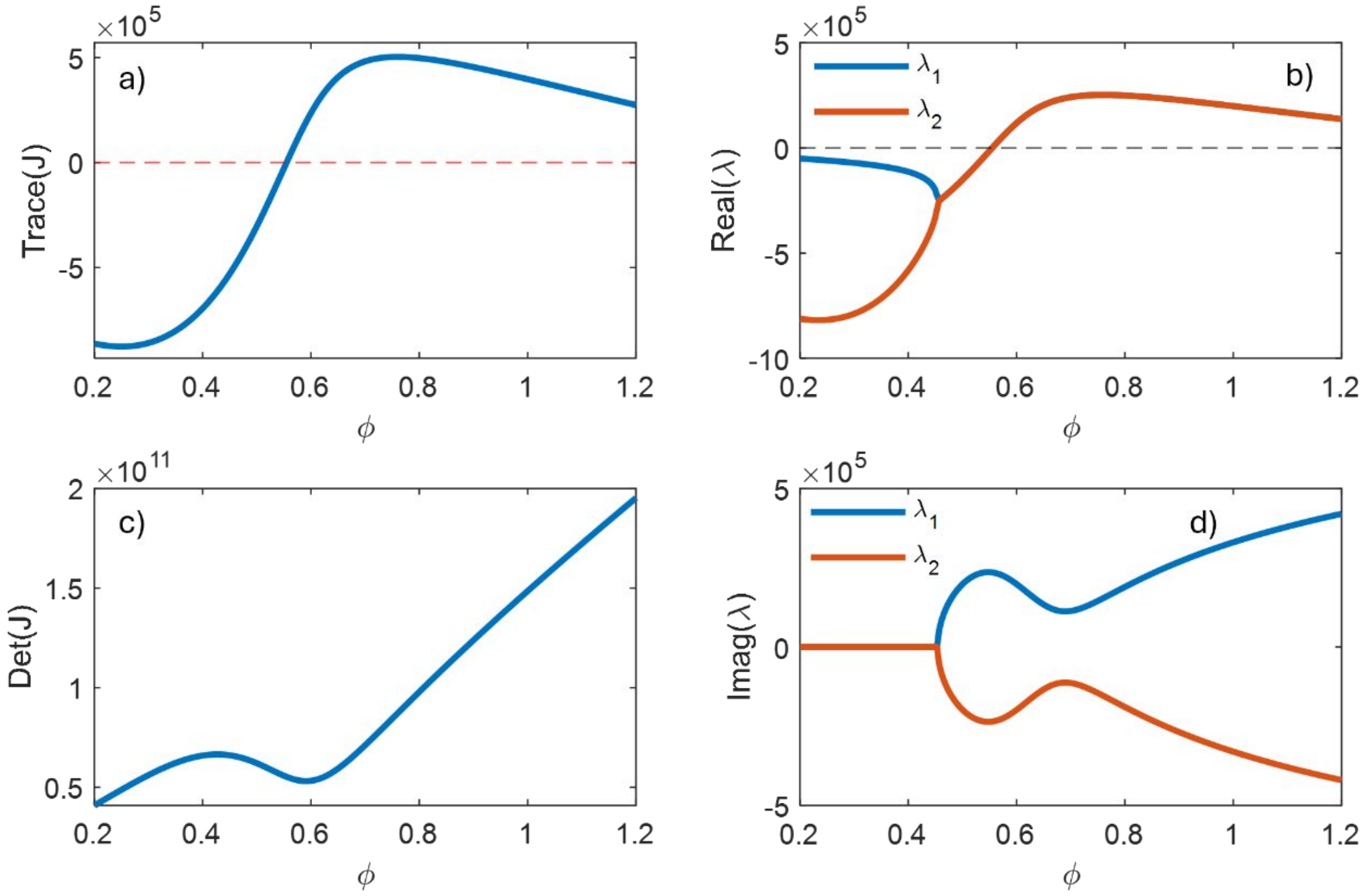

Figure S6. Parameters of the bifurcation analysis of light-induced bursts.
